# Measuring Dietary Botanical Diversity as a Proxy for Phytochemical Exposure

**DOI:** 10.3390/nu13041295

**Published:** 2021-04-14

**Authors:** Henry J. Thompson, Jack O. Levitt, John N. McGinley, Paulette Chandler, Patricia M. Guenther, Inge Huybrechts, Mary C. Playdon

**Affiliations:** 1 Cancer Prevention Laboratory, Colorado State University, Fort Collins, CO 80523, USA; john.mcginley@colostate.edu; 2 Department of Nutrition and Integrative Physiology, University of Utah, Salt Lake City, UT 84112, USA; jack.levitt@utah.edu (J.O.L.); patricia.guenther@utah.edu (P.M.G.); 3 Cancer Control and Population Sciences Program, Huntsman Cancer Institute, Salt Lake City, UT 84112, USA; 4 Department of Medicine, Harvard Medical School & Brigham and Women’s Hospital, Boston, MA 02115, USA; pchandler@bwh.harvard.edu; 5 Nutritional Epidemiology Group, International Agency for Research on Cancer, World Health Organization, CEDEX 08, 69372 Lyon, France; HuybrechtsI@iarc.fr

**Keywords:** botanical diversity, chronic disease risk, gut microbiome, metagenomics, metabolomics, dietary pattern

## Abstract

The study of natural plant molecules and their medicinal properties, pharmacognosy, provides a taxonomy for botanical families that represent diverse chemical groupings with potentially distinct functions in relation to human health. Yet, this reservoir of knowledge has not been systematically applied to elucidating the role of patterns of plant food consumption on gut microbial ecology and function. All chemical classes of dietary phytochemicals can affect the composition of the microbes that colonize the gut and their function. In turn, the gut microbiome affects the host via multiple mechanisms including gut barrier function, immune function, satiety and taste regulation and the activity of biological signaling pathways that influence health and disease. Herein, we report the development of a botanical diversity index (BDI) to evaluate plant food consumption as a novel metric for identifying and quantifying phytochemicals to which an individual is exposed. A rationale is advanced for using the BDI to investigate how plant food diversity impacts gut microbial ecology and functionality.

## 1. Introduction

Major chronic diseases including obesity, type 2 diabetes, cardiovascular disease, stroke, and cancer account for more than 60% of global mortality per annum [1,2]. Evidence continues to emerge that the deregulation of cell signaling pathways underlying the pathogenesis of these diseases, such as chronic inflammation, are interrelated [3]. This provides an avenue for reducing the complexity of preventing and controlling chronic diseases to a common framework. In parallel with understanding shared chronic disease mechanisms, efforts to promote diet and nutrition for chronic disease prevention and control have evolved from a focus on specific nutrients or foods to a recognition of the importance of dietary patterns, which represent the totality of foods and beverages habitually consumed and the interactive, synergistic, and antagonistic nature of dietary exposures on human metabolism and disease [4,5,6]. Food pattern modeling further characterizes amounts and types of foods within a dietary pattern, such as fruits, vegetables, and grains [7]. Recent advances in next-generation sequencing have revealed that the gut microbiome is a key mediator of the effects of food on chronic disease processes; although there is not yet a consensus on either what constitutes a healthy gut microbiome or on which food consumption patterns are most effective in maintaining the functional activity of the gut microbiome [8,9,10,11]. Emerging evidence suggests that plant food diversity may play a role. For example, a study from the American Gut Project citizen science initiative showed that the number of unique plant species eaten was associated with microbial diversity. Short-chain fatty acid fermenters were associated with eating >30 versus <10 plant types per week [12]. We advance the idea that new insights about the interface of diet, the gut microbiome, and chronic disease mechanisms can be gained by considering botanical plant food diversity through the lens of pharmacognosy, which in this context pertains to the study of drug substances of natural origin. This approach has the potential to pave the way to applying a precision medicine framework to the concept of “culinary medicine” with the goal of intervening through diet–gut microbiome interactions to modulate shared chronic disease processes for disease prevention and control [11,13].

## 2. Characterizing Dietary Diversity

Dietary variety, representing the pattern of food and beverage diversity in the diet, has been promoted to help achieve a nutritionally adequate diet [7,14]. Many studies exist on the topic of dietary diversity, using a variety of indices to quantify it in a population setting. Dietary diversity algorithms have tended to count number of servings of dietary guideline-recommended food groups or subgroups consumed in a specified timeframe, with or without penalization according to meeting consumption targets [15,16,17,18,19,20,21,22,23,24]. Some diversity scores consider, in addition to number of food items, the distribution and health value of consumed foods, with higher scores assigned as variation in food intake becomes healthier [25,26]. More quantitatively rigorous capture of dietary diversity has been accomplished with the use of a modified Berry–Simpson index, a common measure of diversity used in ecology and economics [20,27,28]. Such scores account for both the number of foods or food groups consumed and the quantity consumed across these food groups. Functional diversity indices further reflect diversity in nutrient composition of the foods consumed [29], thus additionally capturing nutritional adequacy.

A limitation of existing methods for measuring dietary diversity is that diversity scores can be inclusive of both healthy (nutrient dense) and less healthy (not nutrient dense) food items and may fail to account for proportionality of dietary components. In light of several recent studies [21,26], an American Heart Association science advisory concluded that dietary diversity may be associated with increased energy consumption and obesity [30]. The advisory’s primary recommendation was to focus on increasing diet quality through adequate consumption of plant food, protein sources, low-fat dairy products, vegetable oils, and nuts and limiting consumption of sweets, sugar-sweetened beverages, and red meats, while stating a need for further research into the more specific aspects of dietary variety [30]. These reported concerns provide an opportunity to thoughtfully reconsider how plant food intake patterns are identified.

## 3. Rethinking the Assessment of the Plant Food Components of Dietary Patterns

A common axiom in the nutrition and dietetics community and government nutrition education programs is to “eat a variety of foods”, also communicated as, “eat the rainbow” [31,32]. This traditional recommendation is reflected in the Dietary Guidelines for Americans and other international dietary guidelines and is formulated using culinary definitions of foods (e.g., fruits, vegetables, and grains) [7]. A careful inspection of how these terms are defined and the manner in which they are used reveals a surprising amount of ambiguity about the rationale for grouping foods, especially the subgroups [33]. In the interest of better understanding the benefits of plant food intake, both individually and in combination, on the gut microbiome, the current food groups and subgroups may be insufficiently granular to capture the chemistry that dictates both what microbes are likely to populate the gut and their functional activity. In the following sections, we consider the advantages gained from evaluating diet with a focus on plant food diversity.

### 3.1. Pharmocognosy

**Figure 1:**
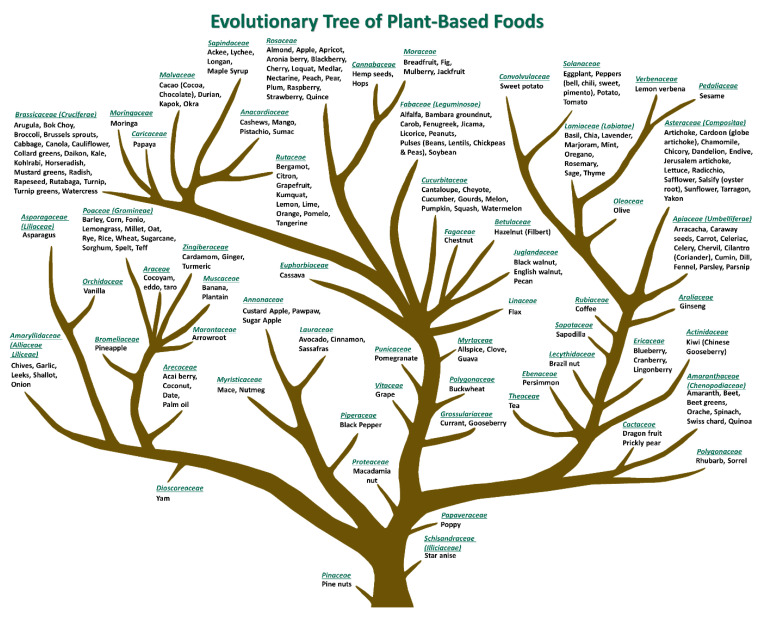
Evolutionary Tree of Plant-Based Foods. Botanical families occupying proximal branches are more chemically similar than those on distant branches. Reprinted with the permission of Elsevier (2021).

The American Society of Pharmacognosy defines pharmacognosy as, “the study of the physical, chemical, biochemical, and biological properties of drugs, drug substances, or potential drugs or drug substances of natural origin as well as the search for new drugs from natural sources”. While the term is not new, first being used in 1841, to our knowledge, leveraging the principles of pharmacognosy to systematically categorize commonly consumed plant foods based on the probability of chemical similarity is novel. The organizational structure that we have developed is illustrated in an updated version of the Evolutionary Tree of Plant-Based Foods (Figure 1) that we originally published [34]. The tree was designed using principles of chemotaxonomy, the classification of plants based on similarities and differences in biochemical composition [35]. Accordingly, the probability for chemical similarities among foods derived from the same botanical family is greater than foods derived from different botanical families [36]. Many potentially significant bioactive phytochemicals remain unidentified. However, botanical groupings may provide direction for emerging technologies like mass spectrometry to identify and quantify compounds linked to biological function, health status, and disease. One such example highlighting the potential of this approach is the finding that consumption of cruciferous vegetables belonging to the botanical family *Brassicaceae* has been linked to reduced risk of chronic diseases and mortality [37]. Glucoraphanin [38], a glucosinolate, serves as an inert precursor to the frequently studied chemoprotective compound sulforaphane. Identification of edible plants containing this metabolite provides a roadmap for identifying similar functional foods or “plants as medicine”.

### 3.2. Phytochemicals

The rationale for developing a novel dietary pattern score for measuring plant food botanical diversity stems from the premise that genetically distinct plant foods, as depicted in the Evolutionary Tree of Plant-Based Foods, contain diverse phytochemicals that may be digested, used as substrate by microbes, absorbed, and metabolized to different extents. Phytochemicals represent the primary and secondary metabolites derived from biosynthetic processes underlying growth, development, and reproduction in every plant [35]. Primary metabolites include carbohydrates, amino acids, lipids, and nucleic acids. They exist in many complex conformations that affect both the ability to digest them to absorbable structures and that also affect the ability of microorganisms to assimilate them as fuel sources [39]. It is estimated that up to 5 g of dietary fat; 25 g of dietary protein, peptides, and amino acids; and 40 g of dietary carbohydrate enter the large intestine each day, affecting the type, amount, and function of the microbes that colonize that intestinal segment (reviewed in [40]). Microbial access to these macromolecules as fuel sources is determined by the primary, secondary, and tertiary structure of their “digested macronutrient”. Plant secondary metabolites fall within a wide range of chemical classes and are estimated to include over 10,000 distinct chemicals (Table 1). Secondary metabolites generally exist in plants bound to other molecules, for example, carbohydrates, lipids, and proteins; and this affects their bioavailability as well as the microbial species that occupy the intestinal tract. Details of the interactions of microbes with both primary and secondary phytochemicals have recently been reviewed [13,40].

### 3.3. Botanical Diversity Index (BDI)

A prior human randomized dietary diversity trial compared diets providing 5 versus 18 botanical families per day and demonstrated the efficacy of botanical diversity for modulating disease-relevant biological pathways including oxidative stress [19]. We propose modeling the diversity of botanical foods consumed as a useful means of examining the complex interaction between diet, microbiome, and host metabolism. This required the development of a novel, quantitative metric to sufficiently capture dietary botanical diversity. We adapted the Berry–Simpson index [41] to create the BDI. The index was derived from a food frequency questionnaire (FFQ). For each line item representing a botanical food, the amount of food reported was converted to cup equivalents (for fruits, vegetables, and legumes) or ounce equivalents (for grains) per day, using the 2017–2018 United States Department of Agriculture (USDA) Food Patterns Equivalents Database (FPED) [42]. Intake of each botanical group was quantified as the square of the number of cup or ounce equivalents consumed per day divided by the total number of botanical families evaluated; the values for each botanical group were then summed, and the result was subtracted from one. Possible values for the index range from 0 to [1–1/n] (n being the number of botanical groups evaluated). Theoretically, a BDI score of 0 would indicate plant food consumption from only one botanical family, while a score of 1 would indicate consumption of all botanical families evaluated.

A limitation of this approach to generating a diversity index is that the number of botanical families represented is limited by the number captured by the dietary assessment method used, as in the case with an FFQ. Twenty-four-hour dietary recalls yield greater granularity and, therefore, may capture more botanical families. On the other hand, they may capture fewer botanical families because the observation period is very short. Multiple 24 h recalls would be needed to model dietary botanical diversity in the diet. Depending on the research question of interest, multiple administrations of a 24 h recall can be used to model usual dietary intake for nutrients and food groups [43]; however, statistical models would need to be developed to estimate the BDI.

An example of the BDI score algorithm is presented in Equation (1).

(1) $$\begin{array}{c} \mathrm{BDS}\;\mathrm{Score}\;[1-\sum s_{i}^{2}]=1\;-\;[(\mathrm{rosaceae}/n{)}^{2}+(\mathrm{musaceae}/n{)}^{2}+(\mathrm{fabaceae}/n{)}^{2}+\\ (\mathrm{amaranthaceae}/n{)}^{2}+(\mathrm{poaceae}/n{)}^{2}+(\mathrm{brassicaceae}/n{)}^{2}+(\mathrm{malvaceae}/n{)}^{2}+\\ (\mathrm{cucurbitaceae}/n{)}^{2}+(\mathrm{apiaceae}/n{)}^{2}+(\mathrm{rubiaceae}/n{)}^{2}+(\mathrm{amaryllidaceae}/n{)}^{2}+\\ (\mathrm{rutaceae}/n{)}^{2}+(\mathrm{vitaceae}/n{)}^{2}+(\mathrm{solanaceae}/n{)}^{2}+(\mathrm{convolvulaceae}/n{)}^{2}+\\ \left(\mathrm{asteraceae}/n{)}^{2}+(\mathrm{bromeliaceae}/n{)}^{2}+(\mathrm{Theaceae}/n{)}^{2}\right] \end{array}$$

n: total number of botanical families represented on the food frequency questionnaire. All values in servings/day.

We calculated the BDI for the menus provided in the randomized dietary trial by Thompson et al., (2006), where study groups were given 8–10 servings of plant foods per day but assigned a low (5 families) or high (18 families) botanical diversity diet over 14 days [44]. The low-diversity menu received a BDI score of 0.76 while the high-diversity menu scored 0.93. Applying the same scoring algorithm to data from a nested study within the Prostate, Lung, Colorectal and Ovarian Cancer Screening Trial (PLCO) that measured dietary intake using a National Cancer Institute food frequency questionnaire, the Diet Questionnaire, Version No. 10/99, ref. [45] yielded a range of scores from 0.34 to 0.89 (mean 0.75 and median 0.77). Average intake of foods according to botanical family is presented in Table 2.

Although the study population was relatively homogenous (i.e., predominantly postmenopausal white women), these results demonstrate the broad range of dietary botanical diversity consumed. We stratified BDI score in the PLCO study by demographic and lifestyle characteristics, finding no differences in the distribution of scores across strata for body mass index, energy intake, or age group; but differences were observed by the number of hours spent per day in vigorous physical activity (Table 3).

## 4. Food Intake Pattern, the Gut Microbiome, and Human Health and Disease

There has been an explosive expansion of the literature on food, the gut microbiome, and human health and disease, with major reviews being published [13,40,46]. It is apparent that increased granularity in the assessment of foods to which the gut is exposed is required to understand microbial ecology and function in relation to human health and disease. The human gut microbiota consists of the 10–100 trillion symbiotic microbial cells harbored by each person, primarily bacteria [47]. These microbiota are phylogenetically diverse, and this gives rise to metabolic heterogeneity. A human microbiome constitutes 3 million non-redundant genes; whereas, the human genome is comprised of approximately 20,000 genes of metabolism [48]. The gut microbiota impact essential functions of the host including gut barrier function, immune function, satiety, and taste regulation and the activity of biological signaling pathways that influence health and disease [46,49,50]. The location of microbiota throughout the intestinal tract is determined by exposure to substrates, including those derived from the diet, and host interactions that promote microbial transcriptional activity (i.e., the meta-transcriptome) with disease-relevant targets [51]. An example is the gut bacterial fermentation of fiber to butyrate, which can lead to direct oncogene suppression in colonocytes and other cancer-preventive effects on inflammation and immune modulation [52,53,54].

The interface of diet with microbial activity is complex. In addition to gut microbial diversity, there is considerable inter-individual variation in nutrient requirements and utilization. Nutritional adequacy and diet-related disease risk are determined by diet exposure, genetic variation in nutrient metabolism, their interaction with the gut microbiome, and the effects on the metabolome and proteome. However, little is known about how dietary botanical diversity influences the interplay between human gene expression, microbial activity, and human metabolism. Diet quality (e.g., as measured by the Healthy Eating Index and Mediterranean Diet Score), on the other hand, has been associated with gut microbial diversity [55,56,57] and hundreds of both endogenous metabolites and products of exogenous food metabolism [58]. The interaction of the gut microbiome with host metabolism via microbial metabolites [59,60] can lead to disease-relevant metabolic alterations such as changes in circulating glucose [61] and cell signaling that can be either beneficial or harmful [62]. Added to this complexity, culinary techniques that modify the chemical composition of food (e.g., preserving (salting, additives), processing, preparing (removing vegetable/fruit skin), and cooking) may alter microbial-food interaction and subsequent phytochemical exposure. To disentangle this complexity with a view to identifying patterns of eating that optimize health, methods to better evaluate phytochemical exposures in the intestinal tract are needed. To this end, the BDI was developed as a tool to measure dietary botanical diversity as a proxy for phytochemical exposure to the human gut microbiome. Superimposing this botanical scoring system on an individual’s omics and meta-omics profiles using deep learning algorithms is a promising approach to support an omics-driven era of precision culinary medicine for health promotion and disease prevention. Once these interactions are mapped, clinical value may be obtained by measuring alpha and beta diversity of gut microbial species or stool metabolites and recommending combinations and diversity of plant foods for re-shaping the gut microbial landscape.

## 5. Final Comments

The BDI is a novel metric for examining the interplay among plant foods, the thousands of dietary phytochemicals to which an individual is exposed, and the human gut microbiome. Considering the suggestive associations between the gut microbiome and human health, this approach has exciting potential to improve understanding of the molecular underpinnings with which plant foods exert their health effects and provides a robust chemical framework for developing personalized dietary guidance.

## Figures and Tables

**Table 1:** Bioactive compounds in each class of plant secondary metabolites.

Chemical Classes	Examples of Bioactive Compounds
Alkaloids	7-Acetylintermedine, 7-Acetyllycopsamine, Anabasine, Anatabine, Atropine, Berberine, Brucine, Caffeine, Capsaicin, Catuabine, Codeine, Coniine, Cytisine, Ecgonine, Emetine, Ephedrine, Ergine, Hydrastine, Hygrine, Morphine, Narceine, Narcotine, Nicotine, Nornicotine, Papaverine, Pelletierine, Pilocarpine, Piperine, Quinine, Sanguinarine, Scopolamine, Seratonin, Sparteine, Strychnine, Symphytine, Thebaine, Theobromine, Trigonelline, Vinblastine, Vincristine
Amines	Piperazine, Piperidine, Pyrrolidine (Tetrahdyropyrrole)
Cyanogenic glycosides	Dhurrin, Laetrile (Amygdalin), Linamarin, Lotaustralin, Prunasin, Sambunigrin, Taxiphyllin, Vicianin
Diterpenes	Dihydrogrindelaldehyde, Dihydrogrindelic Acid, Erythrofordin, Hedychilactone, Hedychinone, labd-13E-en-15-oate, Norerythrofordin, Phytol, Retinoids, Retinol, Taxol
Flavonoids	Apigenin, Baicalein, Biochanin A, Catechin, Coumestrol, Cyanidin, Daidzein, Deguelin, Delphinidin, Epicatechin, Epicatechin, Epigallocatechin, Eriodictyol, Fisetin, Galangin, gallate, gallate, Gallocatechin, Genistein, Glycitein, Hesperidin, Isorhamnetin, Kaempferol, Luteolin, Malvidin, Myricetin, Naringenin, Naringin, Pachypodol, Pelargonidin, Peonidin, Petunidin, Quercetin, Rhamnazin, Rotenone, Rutin, Silymarin, Tangeritin, Wogonin
Glucosinolates	Glucoberteroin, Glucobrassicanapin, Glucobrassicin, Glucocheirolin, Glucoerucin, Glucoiberin, Gluconapin, Gluconapoleiferin, Gluconasturtiin, Progoitrin, Sinigrin
Monoterpenes	Borneol, Camphor, Carene, Carveol, Carvone, Citral, Citronellal, Citronellol, Eucalyptol, Eucalyptol, Geraniol, Limonene, Linalool, Myrcene, α-Pinene, β-Pinene, Terpineol
Non-protein amino acids	Alliin, Butiin, Canavanine, S-Allyl Cysteine, Djenkolic Acid, Ethionine, Etiin, Isoalliin, Methiin, Propiin
Phenylpropanes	Caffeic Acid, Piceatannol, Pterostilbene, Resveratrol, Rosavins, Sesamol, Theaflavin, Thearubigin
Polyacetylenes	Capillin, Dihydropanaxacol, Falcarindiol, Falcarinone, Panaxacol, Panaxydol, Panaxynol (Falcarinol), Panaxytriol
Polyketides	Acetogenins (Annonacin Uvaricin), Aflatoxin, Aloenin, Aloesin, Amphotericin, Anthraquinones, Azithromycin, Barbaloin, Bullatacin, Clarithromycin, Discodermolide, Erythromycin A, Pikromycin, Tetracyclines
Sesquiterpenes	Artemisinin, Bisabolol, Cadinene, Caryophyllene, Copaene, Farnesene, Farnesol, Guaiazulene, Lactucin, Longifolene, Parthenolide, Vetivazulene
Tetraterpenes	Annatto, α-Carotene, β-Carotene, and β-Cryptoxanthin, Crocetin, Crocin, Cryptoxanthine, Lutein, Lycopene, Phytoene, Phytofluene, Sporopollenin, Zeaxanthine
Triterpenes, saponins, steroids	Betulinic Acid, Ginsenosides, Glabrolide, Glycyrrhizin, Lanosterol, Lantadene, Lantanolic Acid, Lantic Acid, Licorice Acid, Liquiritic Acid, Lupeol, Oleanolic Acid, β-Sitosterol, Squalene, SU1, Ursolic Acid

**Table 2:** Mean intake (servings * per day) of botanical families, estimated from the Diet Questionnaire used in the Prostate, Lung, Colorectal and Ovarian cancer cohort (*n* = 354).

Botanical Family	Example Foods	Mean ± SD Servings */day
Poaceae (Gramineae)	Cereals/grains, corn, rice	4.29 ± 2.26
Rubiaceae	Coffee	3.15 ± 4.13
Theaceae	Tea	1.29 ± 2.79
Solanaceae	Potatoes, tomatoes, peppers	2.32 ± 3.5
Rosaceae	Other fruits (apples, pears, apricots, strawberries)	1.74 ± 1.56
Fabaceae (Leguminosae)	Dried beans and peas, peanuts	1.52 ± 2.00
Rutaceae	Citrus fruits	1.36 ± 1.46
Vitaceae	Grapes, raisins	0.78 ± 1.56
Cucurbitaceae	Cantaloupe, cucumber, squash, watermelon	0.76 ± 0.60
Musaceae	Banana	0.58 ± 0.48
Brassicaceae (Cruciferae)	Broccoli, Brussels sprouts, cabbage, kale	0.56 ± 0.44
Asteraceae (Compositae)	Lettuce	0.56 ± 0.42
Apiaceae (Umbelliferae)	Celery, carrots, cauliflower	0.32 ± 0.26
Amaranthaceae (Chenopodiaceae)	Spinach, Swiss chard, beet greens	0.20 ± 0.30
Amaryllidaceae (Alliaceae, Liliaceae)	Garlic, onion	0.14 ± 0.12
Convolvulaceae	Sweet potato	0.06 ± 0.12
Bromeliaceae	Pineapple	0.04 ± 0.08

* 1 serving = 1 oz equivalent of grains, 1 cup of coffee or tea, or ½ cup equivalent of fruit or vegetable.

**Table 3:** Botanical Diversity Index (BDI) stratified by demographic and lifestyle factors in the Prostate, Lung, Colorectal and Ovarian cancer cohort (*n* = 354).

	*N*	Mean ± SD	ANOVA *p*-Value
Daily energy intake (kcal)			
<1500	119	0.76 ± 0.10	0.80
1500–<2000	119	0.75 ± 0.08	
2000–<2500	66	0.76 ± 0.09	
≥2500	50	0.75 ± 0.09	
Hours spent in vigorous physical activity per week			
None	60	0.72 ± 0.09	0.002
<1	64	0.75 ± 0.08	
1	45	0.76 ± 0.09	
2	55	0.76 ± 0.09	
3	52	0.78 ± 0.06	
4+	75	0.76 ± 0.09	
Body mass index (kg/m^2^)			
<25	88	0.76 ± 0.09	0.44
25–<30	121	0.76 ± 0.09	
30+	145	0.75 ± 0.08	
Age (years)			
<55	34	0.77 ± 0.09	0.90
55–<60	83	0.76 ± 0.09	
60–<65	114	0.75 ± 0.09	
65–<70	92	0.76 ± 0.08	
70+	31	0.75 ± 0.09	

## Data Availability

Data from the PLCO cohort is available for analysis with data-only project approval at https://cdas.cancer.gov/plco/, (accessed on 5 March 2021).
